# Bridging gaps in oral health education in a medical school in the United States: a pilot study

**DOI:** 10.1186/s12909-022-03648-5

**Published:** 2022-07-28

**Authors:** Mallory Morse Morel, Elizabeth Chuang, Nadia Laniado

**Affiliations:** 1 Division of Oral and Maxillofacial Surgery, Jacobi Medical Center/Mt Sinai Beth Israel, 1400 Pelham Parkway South, Building 1, Suite 3NE1, Bronx, NY 10461 USA; 2 Family and Social Medicine, Albert Einstein College of Medicine, Montefiore Medical Center, Bronx, NY USA; 3 Dentistry, Epidemiology & Population Health, Jacobi Medical Center, Albert Einstein College of Medicine, Bronx, NY USA

**Keywords:** Oral Health, Interprofessional Education, Curriculum Development

## Abstract

**Background:**

Oral health is an important component of medical education given its connection to overall health and quality of life; however, oral health is infrequently incorporated into medical school curricula in the United States. The aim of this study was to pilot a novel oral health care clerkship for United States medical students that implemented the Smiles for Life (SFL) curriculum, in-person clinical activities, and pre and post curricula assessments to assess knowledge acquisition, attitude change, and clinical skill development.

**Methods:**

Third year medical students at Albert Einstein College of Medicine, Bronx, New York, volunteered (*n* = 37) for a clerkship in oral health. Students completed the Smiles For Life National Oral Health Curriculum and participated in three half-day clinical sessions in a hospital-based dental clinic. The participants were evaluated on knowledge acquisition, attitude change, and clinical skill development through a pre and post clerkship assessment in order to assess the efficacy of the intervention.

**Results:**

There was a 23.4% increase in oral health knowledge (*p* < 0.001) following participation in the online modules and clerkship. Additionally, attitudes in the following domains showed improved familiarity and proficiency: causes and prevention of dental caries (78.4%, *p* < 0.001) and periodontal disease (83.8%, *p* < 0.001), provision of oral health information to patients (67.6%, *p* < 0.001), and ability to conduct an oral examination (62.2%, *p* < 0.001).

**Conclusions:**

Third year medical students who participated in a novel oral health clerkship demonstrated significant increases in basic oral health knowledge and reported increased comfort in providing oral examinations and anticipatory guidance to patients. The results support the feasibility of this approach to incorporating oral health education into a medical school curriculum in the United States.

**Supplementary Information:**

The online version contains supplementary material available at 10.1186/s12909-022-03648-5.

## Background

“Say ah” is probably the most commonly heard refrain in a doctor’s office; yet, most physicians do not feel comfortable with the oral cavity [1]. Many systemic diseases are known to have correlations with diseases of the oral cavity, such as diabetes, coronary artery disease, and Alzheimer’s disease [2–5]. However, currently oral health care and general health care in the United States (U.S.) are delivered in fragmented siloes. Care processes and workflows in the U.S. are compartmentalized between medicine and dentistry, which prohibits cooperation between practitioners and worsens healthcare outcomes for patients [6, 7]. This fragmented United States healthcare delivery model is reflected in the oral health care education given to physicians. Despite growing awareness of the emerging evidence of oral-systemic relationships, surveys have demonstrated that the knowledge base of medical students is deficient with regard to oral health [8].

In 2008, a report of the Association of American Medical Colleges (AAMC) was published that advocated for oral health education for medical students [9]. The AAMC now expects medical students to demonstrate competence in multiple domains within oral health. These domains include understanding the pathophysiology of caries and periodontal disease and its implications for systemic health, performing oral health screenings, promoting preventive strategies, and collaborating with dental professionals. The final educational objective detailed in the AAMC report is to elicit a change in attitude related to the topic of oral health care. The overall objective is to encourage new physicians to incorporate oral health into their daily practice regardless of their specialty.

Some medical schools have attempted to incorporate changes to their curricula to address the AAMC’s charge and incorporate oral health care education. However, in a recent study, it was found that 69.3% of surveyed medical schools reported offering less than 5 hours of oral health curriculum and 10.2% offered zero hours of oral health curriculum [8]. One significant barrier for medical schools to incorporating an oral health curriculum is a lack of awareness of how to effectively design a curriculum and assess its outcome. This is highlighted in a study that found 47.9% of surveyed schools were “uncertain” of what curriculum they would design and deliver if they were tasked with adding more hours of instruction on oral health [8].

One such curricula that exists is the Society of Teachers of Family Medicine’s (STFM’s) Smiles for Life (SFL) evidence-based curriculum [10]. SFL was developed to support the integration of oral health and primary care and is currently the most widely used oral health curriculum for health professionals and includes eight courses and other resources that are available online for free (https://www.stfm.org/teachingresources/curriculum/smilesforlife/). This educational tool was initially developed to address knowledge gaps for primary care providers who are currently practicing or in medical residency. However, other studies have shown success while implementing this curricula to provide oral health education for medical students [11, 12]. One deficiency noted in prior studies is the focus on knowledge acquisition in regards to oral health care without improving or assessing clinical skill, clinical comfort, or practical application of said knowledge. Medical schools that have incorporated skill-based programs report that the incorporation of skill acquisition is a key component to the program’s success [13].

The aim of this study was to pilot a novel oral health care clerkship for United States medical students that implemented the SFL curriculum, in-person clinical activities, and pre and post curricula assessments to assess knowledge acquisition, attitude change, and clinical skill development.

## Methods

Our program was developed as an elective during the pediatric clerkship for third year medical students at Albert Einstein College of Medicine, Bronx, New York. Prior to implementation of this pilot study, all medical students at Albert Einstein College of Medicine were exposed to fifteen minutes of instruction dedicated to oral health through a head and neck physical examination didactic lecture and workshop taught during the second year of medical school. Third year medical students have typically completed the didactic portion of medical school and are in their first year of hands-on clinical training. All students (*n* = 270) beginning their pediatric clerkship in the academic years of 2019–2020 and 2020–2021 were contacted via email and were offered the opportunity to volunteer for the study. Those that agreed to the study were incentivized to complete the study with a $50 Amazon gift card given at completion of all required activities. The program was approved by the Albert Einstein College of Medicine’s Office of Medical Education (OME) and supported by a Grant for Excellence in Medical Education. The study was reviewed and approved by the Albert Einstein College of Medicine Institutional Review Board (IRB) (#2018–9556).

### Pretest

Prior to initiating the SFL modules and clinical experiences, students completed a pretest via Qualtrics software. The pretest assessed knowledge via 21 multiple choice questions about common oral health diseases, normal dental anatomy, and epidemiology of oral health conditions (Table 1). The questions were adapted from questions supplied in the SFL curriculum implementation toolkit; questions were chosen specifically for their relevance to the intersection of medicine and oral health. In addition to knowledge-based questions, students were given seven questions, assessed via a Likert scale, regarding their familiarity with oral health topics, attitudes, and comfort regarding oral health care, providing oral health care education and guidance, and performing oral examinations on patients. These questions were adapted from validated questionnaires utilized by similar articles that assessed provider attitudes and comfort [7].

**Table 1:** Knowledge Assessment Questionnaire with participant responses pre and post intervention

Question (Answer)	Pretest % Correct (# Correct/Total)	Posttest % Correct (# Correct/Total)
What is the most common chronic disease of childhood? (Dental caries)	43.2 (16/37)	94.6 (35/37)
Which condition is associated with periodontal disease? (Preterm labor)	5.4 (2/37)	59.5 (22/37)
Which of the following medications is linked to gingival hyperplasia? (Phenytoin)	94.6 (35/37)	100 (37/37)
Which class of medications is NOT generally associated with decreased salivary flow? (Antibiotics)	70.3 (26/37)	81.1 (30/37)
A patient undergoing chemotherapy for cancer is at risk for which of these oral complications due to the effects of chemotherapy? (Oral mucositis)	73.0 (27/37)	70.3 (26/37)
Which of the following infections is NOT potentially caused by direct extension from a dental source? (Otitis media)	29.7 (11/37)	73.0 (27/37)
What is the suggested common pathway linking chronic periodontitis and conditions such as diabetes, coronary artery disease and adverse pregnancy outcomes? (Inflammation)	73.0 (27/37)	94.6 (35/37)
Which of the following is NOT a mechanism for inter-relationships between oral and systemic disease? (Iatrogenic)	64.9 (24/37)	78.4 (29/37)
What is Early Childhood Caries? (An infectious chronic disease)	2.7 (1/37)	59.5 (22/37)
What is a risk factor for developing Early Childhood Caries? (Caries in siblings or caretakers)	73.0 (27/37)	94.6 (35/37)
Periodontal disease can be clinically distinguished from gingivitis in which of the following ways? (Enlarged pockets at the gum base)	48.7 (18/37)	86.5 (32/37)
Which of the following is NOT a common site for oral cancers? (Hard palate)	73.0 (27/37)	94.6 (35/37)
Which of the following is most likely to lead to poorer oral health in the elderly? (All of the above: Alzheimer’s dementia, coronary artery disease, hypothyroidism)	73.0 (27/37)	73.0 (27/37)
Risk factors for adult caries may include all the following EXCEPT: (A vegetarian diet)	91.9 (34/37)	89.2 (33/37)
Which of the following is NOT a normal age-related tooth change? (Root caries)	78.4 (29/37)	91.9 (34/37)
Which of the following statements concerning xerostomia, or dry mouth, is NOT true? (Xerostomia is rarely a problem for patients wearing complete dentures.)	100 (37/37)	97.3 (36/37)
Which of the following has been implicated in the development of recurrent aphthous ulcers? (Trauma)	13.5 (5/37)	59.5 (22/37)
What is a full complement of adult teeth? (32)	70.3 (26/37)	91.9 (34/37)
A caregiver asks you how many teeth her 3 year old child should have. What would you respond? (20)	43.2 (16/37)	89.2 (33/37)
At what age do teeth typically begin to erupt in children? (3–9 months)	37.8 (14/37)	48.7 (18/37)
Oral cancer is most common in which area of the mouth? (Posterolateral tongue)	51.4 (19/37)	75.7 (28/37)
Mean Score	57.6	81.1
Mean Improvement (95% CI, p-value)	23.4% (19.7% to 27.1%, p < 0.001)	
Effect Size (Cohen’s *d*)	2.37	

The questions were adapted from questions supplied in the SFL curriculum implementation toolkit. Results were analyzed using a matched pair student’s t-test with statistical significant defined as *p* < 0.05. *n* = 37

### SFL modules

Students completed four core modules of the SFL curriculum online. SFL provides a multi-media, case-based interactive platform that delivers information to the learner, and then immediately provides the learner with the opportunity to apply their clinical knowledge in case-based scenarios. These four modules covered the relationship between oral and systemic health, the basics of child and adult oral health, and acute dental problems. Students were able to complete these modules at any time throughout the clerkship utilizing a self-directed learning model.

### Clinical experience

In order to provide an active learning component, students were assigned three half-day sessions in pediatric dental clinics at Jacobi Medical Center Department of Dentistry or at the Rose F. Kennedy Center for Excellence in Developmental Disabilities dental clinic. During these clinical sessions, medical students were provided hands-on peer instruction by pediatric dental residents, oral and maxillofacial dental residents, and dental faculty. Clinical skills practiced in these sessions included oral examinations, caries risk assessments, and periodontal disease risk assessments.

### Posttest evaluation

At the end of the rotation upon completion of all three half-day sessions and the SFL modules, students were given a posttest, which included the same multiple choice and Likert scale questions as the pretest in order to assess knowledge gain and changes in attitudes and comfort regarding clinical skills.

### Data analysis

Differences in outcome measures were analyzed via GraphPad Prism Version 8.2.1 for macOS (GraphPad Software, San Diego, California). Scores on the pretest and posttest assessment were compared using a matched pair student’s t-test, with Cohen’s *d* analysis to measure effect size. For the Likert scale analysis, positive responses of ‘very’ or ‘somewhat’ were pooled and compared with ‘neutral,’ ‘not at all,’ and ‘not very’ responses, and significance testing via Fisher’s exact test was performed with a *P* value < 0.05 used to define statistical significance.

## Results

Overall, 43 students (16% of those contacted) volunteered to complete the oral health care clerkship and 37 students (86%) completed the pretest, SFL modules, and posttest. Due to the COVID-19 pandemic, the in-person clinical component of the study was suspended in March of 2020 for 18 of 37 students (49%).

The posttest results demonstrated a statistically significant improvement in knowledge regarding topics in oral health (Table 1). The average correct responses pretest was 57.7% and the average posttest increased to 81.1%, with a mean improvement of 23.4% (95% confidence interval (CI) 19.7% to 27.1%, *p* < 0.001). The effect size of the intervention was calculated as 2.37, showing a large effect size. Examples of these improvements included being able to identify the correct number of teeth for primary and adult dentition, being able to recognize high risks areas of the oral cavity for oral cancer, and risk factors for poor overall oral health. The full survey and participant performance on each component is available in Table 1.

Our results show that our clerkship improved comfort and familiarity universally across these domains with increased percentages of students indicating they were either “very” or “somewhat” comfortable or familiar across a range of topics, which are individually highlighted below. Students indicated a statistically significant increased familiarity in identifying risks for caries (78.4%, *p* < 0.001) and periodontal disease (83.8%, *p* < 0.001) (Table 2). Additionally, we found that students indicated a statistically significant increased comfort in conducting oral exams (62.2%, *p* < 0.001) and in educating their patients about oral health (67.6%, *p* < 0.001) (Table 2). Our data also show that medical students already had an awareness of the importance of collaboration between primary care professionals and dentists, as 97.3% (36 of 37) of students agreed with this statement in the pretest survey.

**Table 2:** Analysis of student comfort and familiarity with topics in oral health pre and post intervention

Question	Pretest # (%)	Posttest # (%)	p value
Are you familiar with the causes, prevention, and signs of dental caries?			
Positive	7 (18.9)	36 (97.3)	< 0.001
Negative/Neutral	30 (81.1)	1 (2.7)	
Are you familiar with the causes and prevention of periodontal disease?			
Positive	3 (8.1)	34 (91.9)	< 0.001
Negative/Neutral	34 (91.9)	3 (8.1)	
Are you aware of links between tobacco use and oral cancer?			
Positive	34 (91.9)	37 (100)	0.24
Negative/Neutral	3 (8.1)	0 (0)	
Can you recognize risks for oral disease?			
Positive	13 (35.1)	37 (100)	< 0.001
Negative/Neutral	24 (64.9)	0 (0)	
Are you comfortable conducting an oral examination?			
Positive	3 (8.1)	26 (70.3)	< 0.001
Negative/Neutral	34 (91.9)	11 (29.7)	
Are you comfortable providing basic oral health information to patients?			
Positive	7 (18.9)	32 (86.5)	< 0.001
Negative/Neutral	30 (81.1)	5 (13.5)	
How important is it for primary care health professionals to collaborate with dentists?			
Positive	36 (97.3)	37 (100)	> 0.99
Negative/Neutral	1 (2.7)	0 (0)	

Positive responses of ‘very’ or ‘somewhat’ were pooled and compared with negative/neutral responses of ‘neutral,’ ‘not at all,’ and ‘not very.’ Significance testing via Fisher’s exact test was performed with a *p* value < 0.05 used to define statistical significance. *n* = 37

In sensitivity analyses, we examined the effect of the interruption of our study caused by the COVID-19 pandemic and found there was no statistically significant difference in knowledge gains with posttest scores of 78.2% pre-COVID-19 compared 84.1% post-COVID-19, a mean difference 5.9% (95% CI –0.4% to 12.2%, *p* = 0.06) or in answers concerning comfort and familiarity with oral health topics (See Supplementary Table 1, Additional File 1).

## Discussion

Third year United States medical students who participated in our novel pilot oral health clerkship demonstrated significant increases in basic oral health knowledge and reported increased comfort in providing oral examinations and anticipatory guidance to patients. Specifically, students showed increased recognition of patient-specific risk factors for oral diseases such as caries and periodontal disease, increased comfort examining the oral cavity, increased comfort counseling their patients about their oral health and oral health behaviors, and increased comfort providing oral health information to patients and in performing oral examinations.

Other studies that have employed pretest and posttest analyses of their medical learners have used this type of analysis to demonstrate knowledge gains and changes in attitudes and perceptions [14]. In alignment with prior studies of similar design, the novel inclusion of in-person clinical activities helped to both improve students’ comfort with hands-on skills and to provide more concrete real world experiences reflected in changed attitudes and beliefs with regard to oral health care. Additionally, interprofessional collaboration and interprofessional education (IPE) are increasingly prominent goals of the Association of American Medical Colleges (AAMC) and this study included multiple opportunities for such engagement [15].

In the educational sciences literature, there are many studies that employ IPE and demonstrate that it is a valuable tool to increase health professions students’ knowledge of the oral-systemic disease connection [14, 16, 17]. For example, models such as the one reported by Estes et al., in which dental students directly supervised nurse practitioner students, demonstrated improved perceived confidence in oral examination skills. [17] In our study, medical students were supervised by dental faculty and received peer education from pediatric and oral and maxillofacial surgery dental residents. This supervision provided an opportunity for interprofessional collaboration, which has been shown to have a significantly positive influence on students’ attitudes about IPE [18]. Given that the fragmentation of overall health and oral health care is an increasingly pressing issue in the U.S., interprofessional collaboration during training is crucial to set the foundation for optimal patient care outcomes once in clinical practice..

Our study has many strengths including the implementation of an online validated didactic component (SFL curriculum) with a clinical component in a dental clinic for peer education and interprofessional collaboration. Our study’s limitations include the COVID-19 pandemic limiting the in-person active learning component. We believe the in-person learning component is the crucial next step forward in improving oral health knowledge and comfort with the application of oral health knowledge in medical education. However, this difference could not be demonstrated due to the limited sample size of the two groups. In future studies, we will reinstate the in-person learning component and further analyze the ways in which it does or does not augment the clerkship. Once the in-person learning component is reinstated, another limitation to the study can be addressed to implement an assessment of learners’ knowledge and attitudes both after the SFL modules and again after the in-person clinical experiences. This would allow the relative effect of the two interventions to be more directly compared. Our smaller sample size as a pilot study does also represent a limitation; however, given the positive results our study demonstrates, the curriculum committee at our medical school is currently considering incorporation of the SFL modules into the curriculum of all medical students. An additional limitation was the lack of a control group that was not exposed to the intervention. It has been shown that the use of single group, pretest and posttest design can be compromised by regression to the mean and maturation [19]. However, we sought to deliver our intervention to the greatest number of learners as possible, so a control group was not feasible in this pilot study design. Finally, since this study took place in a United States medical school, our findings are only generalizable to United States medical students where predoctoral training is siloed between medicine and dentistry.

Our results support the feasibility of this approach to incorporating oral health education in a United States medical school curriculum. Increased student comfort with real-world skill application highlights the importance of utilizing in-person clinical activities to improve student experiences and knowledge acquisition. Our next steps include implementing a 12-month knowledge retention assessment to evaluate long-term knowledge and skill acquisition. The goal will be to use the lessons learned from this pilot study and feedback from participants to expand our program and involve all medical students at our current institution. Finally, we aim to disseminate this approach to serve as a model for implementing oral health curricula at medical schools across the United States.

## Conclusions

In summary, our model of a novel oral health clerkship for third year medical students led to significant increases in basic oral health knowledge and increased comfort in providing oral examinations and anticipatory guidance to patients. An approach that incorporates self-directed learning, in-person clinical experience, and peer interprofessional education can be used to incorporate oral health education in a medical school curriculum in the United States. This study represents a crucial step towards addressing the gap in oral health knowledge evident in medical students and physicians in the United States.

## Supplementary Information

**Additional file 1.** Posttest analysis of student comfort andfamiliarity with topics in oral health pre (*n*=19) and post COVID-19 (*n*=18). Tableand analysis of responses to survey questions pre and post COVID-19.

**Additional file 2.** Study Flow Diagram of Participants. Flow diagram of participants’ path through the studyprotocol, highlighting the division of students requiring protocol modificationdue to the COVID-19 pandemic.

## Data Availability

The full datasets used and/or analysed during this study are available from the corresponding author on reasonable request.

## Abbreviations

AAMC: Association of American Medical Colleges
STFM’s: Society of Teachers of Family Medicine’s
SFL: Smiles for Life
CI: Confidence Interval
OME: Office of Medical Education
IRB: Institutional Review Board
GEME: Grant for Excellence in Medical Education
