# Supplementary material for: Bridging gaps in oral health education in a medical school in the United States: a pilot study
Source: BMC Med Educ. 2022 Jul 28;22:578. doi: 10.1186/s12909-022-03648-5 (PMC9330983; doi:10.1186/s12909-022-03648-5)
Supplement: Supplementary file 1 — Additional file 1. Posttest analysis of student comfort andfamiliarity with topics in oral health pre (n=19) and post COVID-19 (n=18). Tableand analysis of responses to survey questions pre and post COVID-19. [file 12909_2022_3648_MOESM1_ESM.docx]

**Supplemental Table 1:** Posttest analysis of student comfort and familiarity with topics in oral health pre (n=19) and post COVID-19 (n=18).

| Question | Pre-COVID # (%) | Post-COVID # (%) | p value |
| --- | --- | --- | --- |
| Are you familiar with the causes, prevention, and signs of dental caries? |  |  |  |
| Positive | 18 (94.7) | 18 (100) | >0.99 |
| Negative/Neutral | 1 (5.3) | 0 (0) |  |
| Are you familiar with the causes and prevention of periodontal disease? |  |  |  |
| Positive | 17 (89.5) | 17 (94.4) | >0.99 |
| Negative/Neutral | 2 (10.5) | 1 (5.6) |  |
| Are you aware of links between tobacco use and oral cancer? |  |  |  |
| Positive | 19 (100) | 18 (100) | >0.99 |
| Negative/Neutral | 0 (0) | 0 (0) |  |
| Can you recognize risks for oral disease? |  |  |  |
| Positive | 19 (100) | 18 (100) | >0.99 |
| Negative/Neutral | 0 (0) | 0 (0) |  |
| Are you comfortable conducting an oral examination? |  |  |  |
| Positive | 12 (63.2) | 14 (77.8) | 0.48 |
| Negative/Neutral | 7 (36.8) | 4 (22.2) |  |
| Are you comfortable providing basic oral health information to patients? |  |  |  |
| Positive | 15 (78.9) | 17 (94.4) | 0.34 |
| Negative/Neutral | 4 (21.1) | 1 (5.6) |  |
| How important is it for primary care health professionals to collaborate with dentists? |  |  |  |
| Positive | 19 (100) | 18 (100) | >0.99 |
| Negative/Neutral | 0 (0) | 0 (0) |  |

The in-person clinical component was not part of the intervention in post COVID participants. Positive responses of ‘very’ or ‘somewhat’ were pooled and compared with negative/neutral responses of ‘neutral,’ ‘not at all,’ and ‘not very.’ Significance testing via Fisher’s exact test was performed with a p value < 0.05 used to define statistical significance.
