# Supplementary figures and images for: Bridging gaps in oral health education in a medical school in the United States: a pilot study
Source: BMC Med Educ. 2022 Jul 28;22:578. doi: 10.1186/s12909-022-03648-5 (PMC9330983; doi:10.1186/s12909-022-03648-5)

## Additional File 2: Study Flow Diagram of Participants

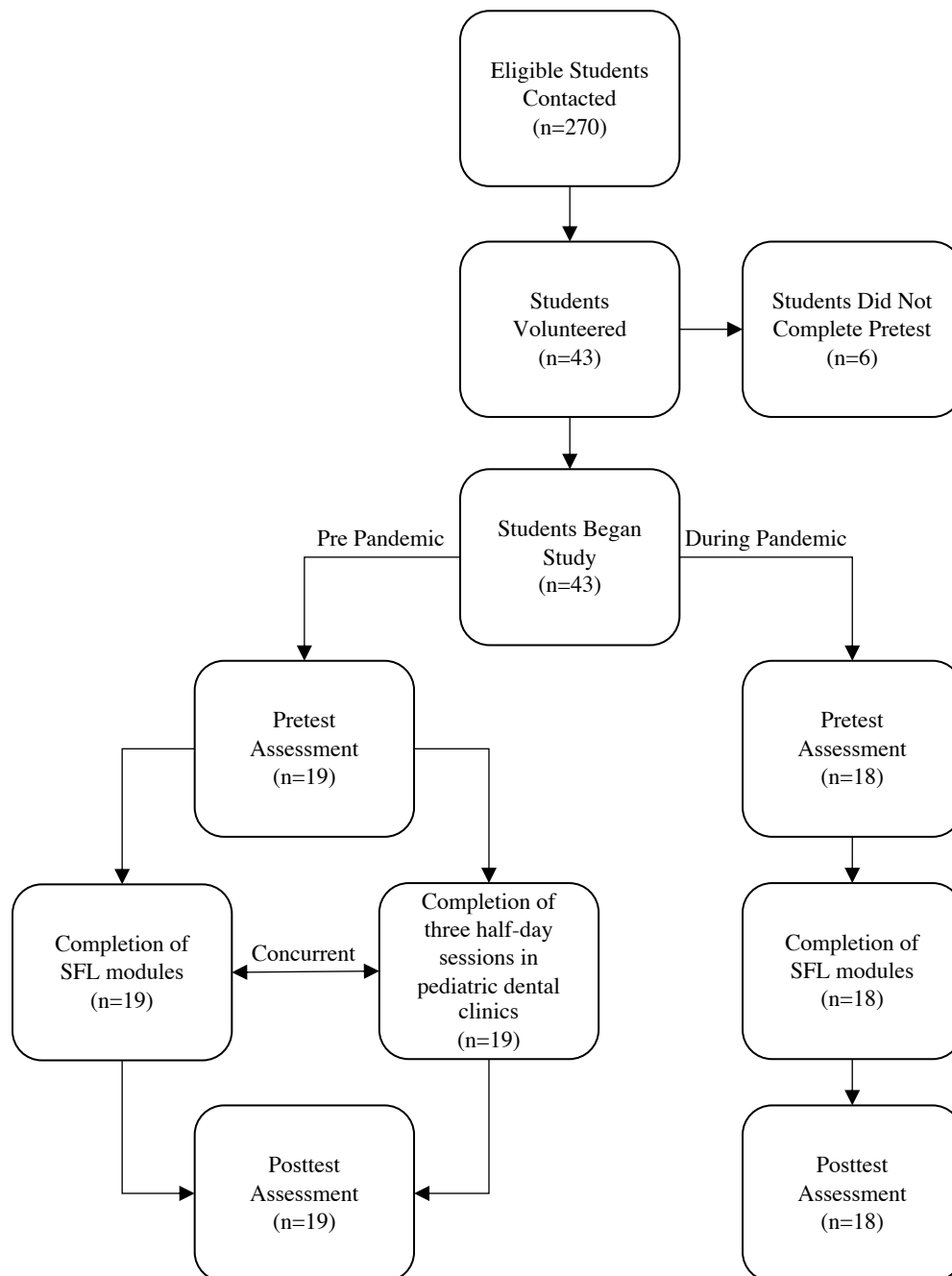

Supplement: Supplementary file 2 — Additional file 2. Study Flow Diagram of Participants. Flow diagram of participants’ path through the studyprotocol, highlighting the division of students requiring protocol modificationdue to the COVID-19 pandemic. [file 12909_2022_3648_MOESM2_ESM.pdf]
